# ScRDAVis: An R shiny application for single-cell transcriptome data analysis and visualization

**DOI:** 10.1371/journal.pcbi.1013721

**Published:** 2025-11-13

**Authors:** Sankarasubramanian Jagadesan, Chittibabu Guda

**Affiliations:** 1 Department of Genetics, Cell Biology and Anatomy, University of Nebraska Medical Center, Omaha, Nebraska, United States of America; 2 Center for Biomedical Informatics Research and Innovation, University of Nebraska Medical Center, Omaha, Nebraska, United States of America; Korea Advanced Institute of Science and Technology, KOREA, REPUBLIC OF

## Abstract

Single-cell RNA sequencing (scRNA-seq) technology has revolutionized biological research by enabling a through exploration of cellular heterogeneity. However, the complexity of data processing pipelines and the need for programming expertise create barriers for many biologists to explore scRNA-seq data. To address this, we developed Single-cell RNA Data Analysis and Visualization (ScRDAVis), an interactive, browser-based R Shiny application tailored for biologists with no programming expertise. ScRDAVis integrates widely used analysis packages, such as *Seurat*, *CellChat*, *Monocle3*, *clusterProfiler* and *hdWGCNA* to provide a user-friendly interface for single-cell data analysis. The application supports single-sample, multiple-sample and group-based analyses, along with features such as marker discovery, cell type annotation, subclustering analysis, and advanced functional studies. Key functionalities include cell-cell communication analysis, trajectory and pseudotime inference, pathway enrichment analysis, weighted gene co-expression network analysis (WGCNA), and transcription factor (TF) regulatory network analysis. ScRDAVis stands out as the first GUI-based platform offering hdWGCNA for co-expression network and TF regulatory network analysis using scRNA-seq data. ScRDAVis provides publication-ready visualizations with data download options in different formats empowering researchers to extract meaningful biological insights and democratizing the analytical capabilities required to comprehensively analyze scRNA-seq studies. ScRDAVis can be freely downloaded from GitHub at https://github.com/GudaLab/ScRDAVis or accessed from any browser at https://www.gudalab-rtools.net/ScRDAVis.

## Author summary

Single-cell RNA sequencing (scRNA-seq) has transformed our ability to study cellular heterogeneity, but its analysis requires integrating many computational steps that are often distributed across multiple tools. To address this challenge, we developed ScRDAVis that can be accessed via a web browser or installed locally on a desktop. This versatile tool unifies nine analytical modules, covering preprocessing, clustering, marker identification, functional enrichment, cell–cell communication, trajectory inference, and regulatory network analysis. We designed ScRDAVis to be very user-friendly, specifically, for those without prior programming expertise. This tool generates interactive visualizations at each step that can be downloaded in seven different formats. Similarly, we provide contextual help menus to document parameter descriptions, default values, and recommended ranges, ensuring reproducibility and transparency. We demonstrate the utility of ScRDAVis with a case study using the publicly available dataset GSE266873. This example illustrates how the integrated modules work together in a cohesive workflow to generate meaningful biological insights. By combining breadth, accessibility, and transparency, ScRDAVis enables a broad community of researchers to explore and effectively interpret single-cell transcriptomics data.

## Introduction

Single-cell RNA sequencing has revolutionized our ability to analyze gene expression at a single cell resolution and opened up new opportunities to study biology using a different lens and gain insights into the cellular heterogeneity, developmental trajectories, and the healthy and disease states of single cells [1,2]. scRNA-seq technology has been pivotal in identifying rare cell types, understanding cell-state transitions, and constructing cellular atlases for organisms [3]. Moreover, the integration of scRNA-seq with scMultiome and spatial transcriptomics approaches further enhances its potential to provide a holistic understanding of cellular functions and interactions within their native tissue context [4]. By uncovering cellular complexity at an unprecedented scale, scRNA-seq is reshaping our understanding of biology, offering new opportunities for biomarker discovery, addressing drug resistance, and making therapeutic interventions [5].

In recent years, advancements in scRNA-seq data analysis tools and web-based platforms have enabled high-throughput data processing, interactive visualization, and functional annotation. Key Tools for scRNA-seq analysis include *CellRanger* from 10x Genomics [6] that processes raw scRNA-seq data from 10x Genomics platforms, including read alignment, barcode processing, and transcript quantification. A popular tool, *Seurat* [7–10], is based on a comprehensive R package for clustering, visualization, and differential expression analysis of scRNA-seq data. *Scanpy* [11], on the other hand, is a scalable Python-based library designed for analyzing large scRNA-seq datasets with efficient computation and integration capabilities. Tools such as *Monocle3* facilitate pseudotime analysis, trajectory inference, and dynamic cellular process studies [1], while *Harmony* [12] and *BBKNN* [13] allow for batch effect correction in integrated single-cell datasets from different experiments. While these command-line tools are powerful, they often require bioinformatics expertise or programming knowledge in R or Python languages, thereby limiting their wider usage by biologists. To address this gap, graphical user interfaces (GUIs) like Cell Ranger Loupe Browser (10x Genomics) and lightweight tools such as *ShinyCell* [14], *Cellxgene* [15], Asc-Seurat [16] and *Single Cell Explorer* [17] were developed to provide basic data exploratory capabilities, yet these tools lack advanced capabilities offered by command-line tools such as trajectory and pathway analyses, and cell-cell communication and Co-expression network analyses (Table 1). A recent tool, ICARUS v3 [18], provides advanced analytical features, including cell–cell communication and trajectory analysis. However, it lacks several other important functions, such as identifying conserved markers between clusters, detecting markers between specific clusters, performing condition-based analysis (for two groups), subclustering-based analysis, GSEA analysis, co-expression analysis using WGCNA, and transcription factor regulatory network analysis.

**Table 1:** Comparison of ScRDAVis and other popular single cell data analysis tools.

Functionality Module	ShinyCell	cellxgene	Single Cell Explorer	Asc-Seurat	Loupe browser	ICARUS_v3	ScRDAVis
**Input file formats**							
H5 file	✔	✔	✔	✔	✔	✔	✔
Seurat Object	✔		✔	✔		✔	✔
Barcode, matrix and feature files					✔		✔
Single matrix file						✔	✔
**Modules**							
Support multiple groups				✔			✔
QC filtering				✔	✔	✔	✔
Normalization and PCA				✔	✔	✔	✔
Doublet identification						✔	✔
Marker Identification/ differential expression	✔	✔	✔	✔	✔	✔	✔
UMAP Visualization	✔	✔	✔	✔	✔	✔	✔
t-SNE Visualization	✔		✔	✔	✔	✔	✔
Heatmap for expressed genes	✔	✔	✔	✔	✔	✔	✔
Conserved markers between clusters				✔	✔		✔
Markers between specific clusters				✔	✔		✔
Bubble, Feature, Violin plot, Ridge plot	✔	✔	✔	✔	✔	✔	✔
Cell type prediction						✔	✔
Condition-based analysis (for two groups)				✔	✔		✔
Subclustering					✔		✔
Correlation Network Analysis							✔
Genome Ontology		✔				✔	✔
Pathway analysis			✔			✔	✔
GSEA analysis							✔
Cell-cell communication						✔	✔
Trajectory analysis						✔	✔
Co-expression using WGCNA							✔
Transcription factor regulatory network analysis							✔
**File export options and user interface**							
Export image files in multiple formats	✔		✔	✔	✔	✔	✔
CSV files	✔		✔	✔	✔		✔
Export Seurat object				✔		✔	✔
command-line/GUI	✔			✔	✔	✔	✔
Web interface					✔		✔
Language/platform	R	Python	Python	R/ Docker	Java/ Phyton	R	R

To address the aforementioned challenges and empower users with limited programming skills to utilize command-line tools, we developed ScRDAVis, a user-friendly bioinformatics tool built on R Shiny applications. ScRDAVis seamlessly integrates leading bioinformatics tools such as *Seurat*, *Monocle3*, *CellChat* [19], and *hdWGCNA* [20]. It supports advanced analyses such as co-expression network construction with *hdWGCNA*, TF regulatory network analysis, trajectory and pseudotime inference, cell-cell communication analysis and pathway and gene set enrichment analysis (*GSEA*) [21]. Its dual availability as a web-based or locally installable standalone software ensures its accessibility to a broad-based researcher community. By streamlining scRNA-seq analysis end-to-end, ScRDAVis empowers users to explore single cell transcriptomics data from different conditions to gain data-driven insights into cellular biology. Here, we present the design and implementation of ScRDAVis, highlight its functionality, and demonstrate its utility using real-world datasets.

## Materials and methods

ScRDAVis is developed in R and utilizes the Shiny framework for interactive functionality. The tool requires R (version 4.5.1 or later), RStudio (version 2025.05.1 or later), *Bioconductor* (version 3.21 or later), and *Shiny* (version 1.11.1 or later) for its optimal performance. ScRDAVis can be accessed via our web server or installed on a local desktop from GitHub. To launch the R Shiny graphical interface on a desktop, users can run the following command in R: shiny::runGitHub(“ScRDAVis”, “gudalab”). Alternatively, the tool can be used online at https://www.gudalab-rtools.net/ScRDAVis. ScRDAVis has been tested on Linux (RedHat Enterprise License and Ubuntu) and Windows (10 and 11) systems. A detailed user’s manual is available at https://www.gudalab-rtools.net/ScRDAVis under the “Manual” tab.

### Data formats and testing

ScRDAVis was tested with publicly available datasets from NCBI in various formats, including: H5 File: GSE271107 [22]; Matrix Files (Matrix, Barcode, and Feature Files): GSE266873 [23]; Seurat Object: GSE250488 [24]; and Matrix Count File: GSE155953 [25]. Users can upload and analyze their own scRNA-seq data in any of the supported file formats, with a file size limit of 2GB on the web platform. For local installations, there is no file size restriction. In the development of our tool and its documentation, we utilized the publicly available dataset GSE277476 in the matrix file format. The images and visualizations showcased in this manuscript were generated using data from this dataset, demonstrating the ease of use and superior capabilities of ScRDAVis in analyzing and interpreting real-world scRNA-seq data. Once a user uploads their data through the web browser, the ScRDAVis R Shiny application generates a unique temporary directory to initiate the analysis process. The workflow begins only after the user clicks *Submit*, at which point the application executes the selected tasks. Upon completion of the analysis, all raw data and processed results are automatically and permanently deleted as soon as the user closes the browser tab in which ScRDAVis is running. Consequently, no user data are retained after the session ends. To repeat an analysis, users must restart the process by re-uploading their data. In addition, ScRDAVis is also available for local installation. Users can download and install the tool on their personal computer, allowing them to perform the analysis entirely on their local system without uploading data to the web-based platform.

### Modules and workflow

ScRDAVis offers nine distinct modules for scRNA-seq analysis. Module 1 provides a guided, step-by-step workflow incorporating Seurat, *SCTransform* [26], and *DoubletFinder* [27] for preprocessing. Once Module 1 is completed, users can freely explore the remaining modules (2–9) in any order based on their analytical needs. ScRDAVis integrates several widely used bioinformatics packages, including Seurat for preprocessing, clustering, and visualization; Monocle3 for trajectory and pseudotime analysis; CellChat for cell-cell communication studies; *clusterProfiler* [28] and *ReactomePA* [29] for Gene Ontology (GO) and pathway enrichment; *fgsea* [30](Korotkevich et al., 2019) for gene set enrichment analysis; *SingleR* [10], *ScType* [31] and *GPTCelltype* [32] for cell type annotation; and *hdWGCNA* for weighted gene co-expression and TF network analysis. This robust integration ensures compatibility with established scRNA-seq workflows, enabling comprehensive and flexible analyses. A complete list of packages used in ScRDAVis is provided in S1 Table.

### Downloadable plots and tables

Summary tables are displayed using the *DataTables* (DT) package, allowing up to 100 rows to be viewed interactively, while the complete tables can be downloaded as.csv files. Graphical plots are available in seven different formats (JPG, TIFF, PDF, SVG, BMP, EPS, and PS), leveraging the *ggsave* and *downloadHandler* functions for easy export. In addition, users can customize the figure dimensions by adjusting the height and width up to 49 inches, as well as set the resolution within a range of 72–300 dpi. These options ensure flexibility in preparing figures that meet the diverse formatting requirements of different journals.

## Results

**Fig 1:**
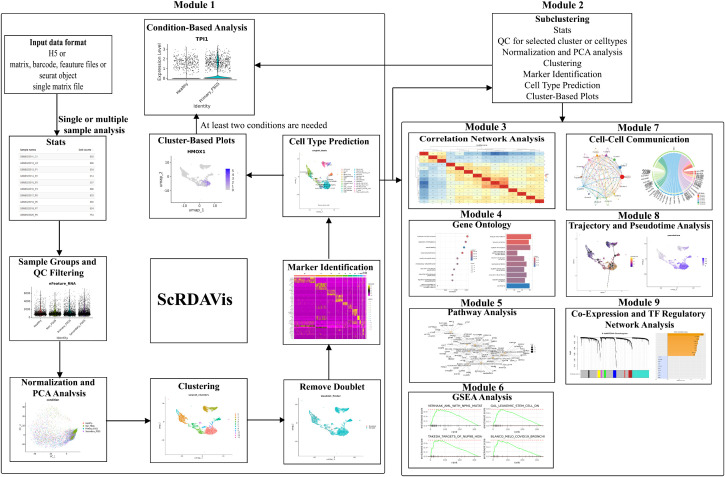
Overview of ScRDAVis workflow. The schematic representation of the ScRDAVis workflow showcases its nine comprehensive modules. The workflow illustrates the sequential steps, inputs, outputs, and the interconnectivity of the modules.

ScRDAVis comprises nine comprehensive modules that facilitate various aspects of scRNA-seq analysis: 1. Single or Multiple Samples Analysis, 2. Subclustering, 3. Correlation Network Analysis, 4. Gene Ontology (GO) Analysis, 5. Pathway Analysis, 6. GSEA Analysis, 7. Cell-Cell Communication Analysis, 8. Trajectory and Pseudotime Analysis, 9. Co-Expression and TF Analysis. All adjustable parameters used in the ScRDAVis web application are detailed in S2 Table. In addition, a contextual help menu is available in each tab and subtab of the interface, providing users with parameter descriptions, default values, and recommended ranges to guide optimization. The supplementary table includes every user-configurable option across analytical modules, with the corresponding default value and a brief functional description. An estimated runtime for ScRDAVis using Windows or Linux servers with a minimum of 32GB RAM was also provided in S3 Table. The detailed workflow of ScRDAVis is illustrated in Fig 1. To demonstrate ScRDAVis capabilities, we analyzed a publicly available scRNA-seq dataset (GSE266873) consisting of 9 samples across three groups Group1 (n = 3, 0–6 hours post-ICH, G1), Group2 (n = 3, 6–24 hours post-ICH, G2), and Group3 (n = 3, 24–48 hours post-ICH, G3).

### Module 1: Single or multiple sample analysis

This module allows users to analyze single or multiple samples and groups, leveraging tools such as *Seurat*, *SCTransform*, and *DoubletFinder*. Key features include:

#### Preprocessing and QC filtering.

**Fig 2:**
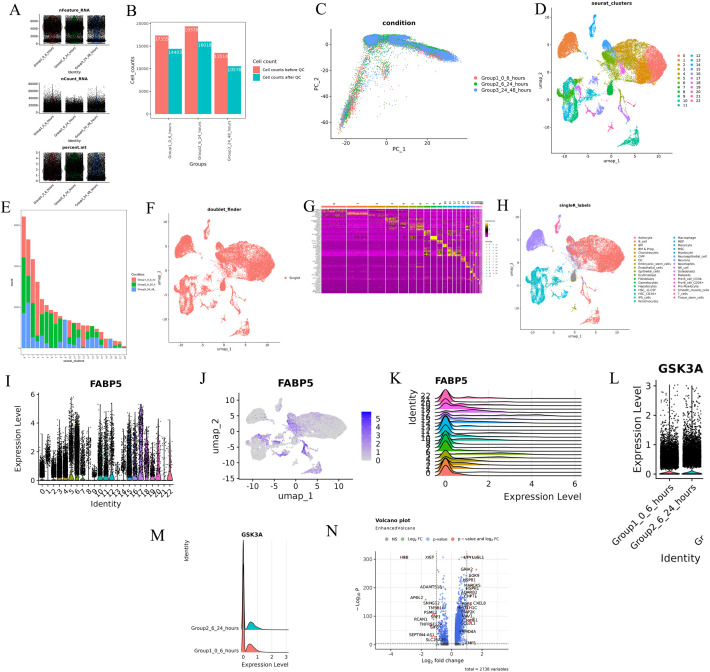
Single or multiple sample analysis. **A)** Quality control (QC) metrics visualized as plots showing cell counts, gene counts, and mitochondrial percentages. **B)** Bar plot represents the cell count based on condition. **C)** Principal component analysis (PCA) plot for dimensionality reduction. **D)** UMAP/t-SNE visualizations of clusters representing cellular heterogeneity. **E)** Bar plot represents the cell counts for each cluster. **F)** UMAP/t-SNE plots after doublet detection and removal. **G)** Heatmap showing marker genes identified for each cluster. **H)** UMAP visualization with cell type annotations based on ScType, SingleR, GPTCelltype, or own annotation. **I-K)** Violin plot, feature plot, and ridge plot illustrating gene expression patterns across clusters. **L-N)** Condition-based differential expression analysis visualized using violin, ridge and volcano plots.

Users begin with preprocessing and quality control (QC) steps assessing sample quality using QC plots and tables and summarizing metrics like cell counts, gene counts, and mitochondrial percentages (Fig 2A and 2B). Filters can be applied to remove low-quality cells at this stage ensuring robust data for downstream analyses.

#### Normalization and dimensionality reduction.

ScRDAVis implements two widely used normalization strategies LogNormalize and SCTransform, to mitigate technical variability before feature selection and projection. By default, LogNormalize performs library-size normalization to a scale.factor of 10,000 followed by a log1p transform, whereas SCTransform applies regularized negative binomial regression to stabilize variance. After normalization, highly variable features are identified using Seurat’s vst method with 2,000 genes by default, and the data are scaled across these features. Principal component analysis (PCA) is then computed on the variable features. ScRDAVis uses 50 PCs by default (user-configurable and recorded in the analysis log for reproducibility). To help users make a data-driven choice of dimensionality, ScRDAVis integrates a JackStraw workflow: users can run JackStraw resampling (default num.replicate = 100) and ScoreJackStraw across the first 1:20 PCs (configurable), visualize JackStrawPlot, and select PCs based on per-PC significance (e.g., p < 0.05). The selected PC range is propagated automatically to downstream steps and written to the output object, and an Elbow plot is included as a heuristic (Fig 2C).

#### Clustering.

After normalization and PCA, ScRDAVis constructs a shared nearest-neighbor (SNN) graph with FindNeighbors using the user-selected PCs and performs graph-based clustering with FindClusters (default resolution = 0.5, adjustable). Low-dimensional embeddings are then computed with UMAP (default n.neighbors = 20, dims = 30, min.dist = 0.3) or t-SNE (default perplexity = 30) on the same feature space, enabling clear visualization of cellular heterogeneity (Fig 2D, 2E). For multi-sample analyses, the tool supports several integration workflows, and by default, sets the recommended reduction and distance metric for each method: under default LogNormalization (no integration), neighbors are computed in PCA space with Euclidean distance; with HarmonyIntegration, in harmony space with cosine distance; with CCAIntegration, in cca space with Euclidean distance; with RPCAIntegration, in rpca space with Euclidean distance; and with JointPCAIntegration, in jointpca space with Euclidean distance. Clustering is performed using Seurat’s clustering algorithm, with options for integration methods like Harmony, CCA, RPCA, and JointPCA. Results are visualized using UMAP or t-SNE plots to depict cellular heterogeneity (Fig 2D and 2E). Cell counts are displayed in bar charts and tables, providing a quantitative summary.

#### Doublet detection and removal.

*DoubletFinder* is integrated to identify doublets, which can introduce noise in scRNA-seq data. Users can filter doublets and visualize updated UMAP/t-SNE plots showing singlet and doublet cells (Fig 2F). Upon removal of doublets, the tool regenerates clusters, updating corresponding tables and visualizations.

#### Marker gene identification.

Marker gene analysis is an essential component in understanding cell types and functions. ScRDAVis supports three methods for marker identification from the Seurat package: *FindAllMarkers* identifies markers for each cluster; *FindMarkers* identifies markers for specific clusters or between clusters; and *FindConservedMarkers* identifies markers conserved across clusters. The output formats of results can be customized and presented as heatmaps (Fig 2G) and tables, offering insights into cluster-specific markers.

#### Cell type prediction.

ScRDAVis simplifies cell type annotation with multiple prediction options such as *ScType* for selecting from 15 tissue-specific datasets, *SingleR* for selecting comprehensive reference datasets for annotation, *GPTCelltype* for AI-driven prediction leveraging GPT models (requires API key for local use). Similarly, the *Custom labels* option allows users to upload their own annotations. Predicted cell types are displayed on UMAP/t-SNE plots (Fig 2H) and using summary tables. In subsequent analyses or in other Modules 2–9, users have the option to work with Seurat cluster predicted cell types from tools such as ScType, SingleR, GPTCelltype, or their own custom-defined labels.

#### Cluster-based visualization.

ScRDAVis offers various options to visualize gene expression results such as *Dot Plots* to summarize gene expression across clusters, *Violin Plots* to visualize distributions of gene expression (Fig 2I), *Feature Plots* to map gene expression spatially on UMAP/t-SNE (Fig 2J), and *Ridge Plots* to display gene expression profiles across clusters (Fig 2K). Users can select the top genes or input the custom gene lists, and plots can also be split by condition or sample for comparisons.

#### Condition-based differential expression analysis.

Users can compare gene expression patterns across conditions to gain deeper insights into biological phenomena. ScRDAVis supports customizable differential expression analysis between two conditions with adjustable parameters such as cell percentage, log fold-change threshold, and type of statistical test. The tool generates a gene expression table for the selected groups, and results are visualized using dot plots, violin plots, ridge plots, feature plots, and volcano plots (Fig 2L–2N).

### Module 2: Subclustering

The subclustering module in ScRDAVis enables users to explore specific clusters in greater detail by refining their analysis at the subcluster level. This module provides the same analytical workflow as Module 1 but is tailored for subclusters. It allows for in-depth exploration of specific cell populations within the dataset. Users can choose one or multiple Seurat clusters, previously predicted annotated labels, or select genes of interest to extract cells for reclustering (positive selection) or exclude genes expressed in cells and perform the analysis using the remaining cells (negative selection). Similar to Module 1, this module also generates comprehensive results, including interactive tables summarizing QC and clustering results, high-quality visualizations such as UMAP/t-SNE plots, heatmaps, and expression plots for detailed analysis of subcluster dynamics.

### Module 3: Correlation network analysis

This module provides insights into gene-gene relationships within clusters or across cell types by constructing correlation networks. This module is particularly useful for comparative exploration of co-expression patterns, understanding cellular functions, and identifying potential regulatory relationships. Users can calculate pairwise correlations using standard statistical methods such as Pearson, Spearman, or Kendall using the R packages *genesorteR* [33] and *ggraph*. Outputs include pairwise correlation values as a matrix between the clusters in the heatmap (S1A Fig), the correlation network (S1B Fig), and the summary table with correlation coefficients for all clusters.

### Module 4: GO analysis

**Fig 3:**
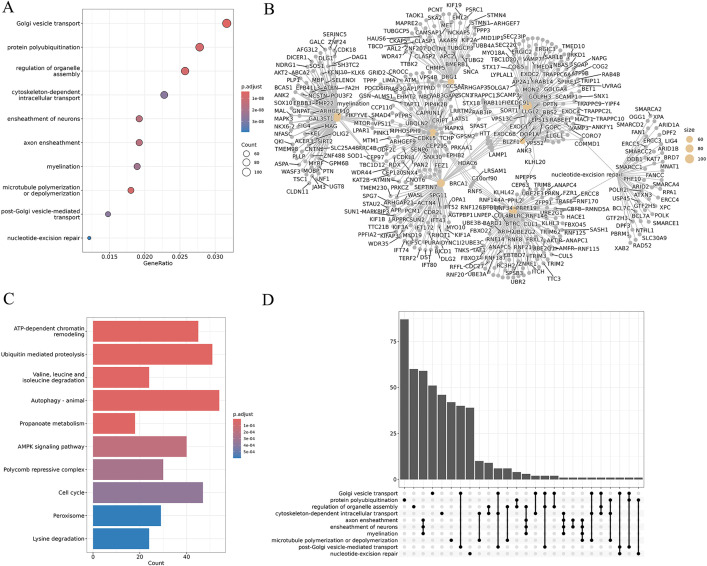
GO analysis. **A**) Dot plot ranking enriched GO terms by p-value and gene ratio. **B)** Network plot illustrating relationships between enriched GO terms and shared genes. **C)** Bar plot highlighting the top 10 enriched GO terms. **D)** UpSet plot shows overlapping genes across multiple GO terms.

The gene ontology module facilitates annotation of the biological roles, molecular functions, and cellular components associated with gene expression patterns in scRNA-seq datasets. Users can annotate single or multiple clusters and separately, provide a custom gene list for analysis using Module 4. The module supports various model organisms, including human, mouse, rat, pig, and rhesus monkey. To perform GO analysis, users can select the clusters of interest from a dropdown menu, and the results are presented using multiple visualization options. A dot plot ranks enriched terms based on p-value and gene ratio (Fig 3A), a network plot illustrates relationships between enriched terms and shared genes (Fig 3B), while a bar plot highlights the top enriched GO terms (Fig 3C). On the other hand, UpSet plot visualizes overlapping genes across multiple GO terms (Fig 3D). Results can also be downloaded using a comprehensive summary table containing GO term IDs, descriptions, enrichment scores, adjusted p-values, and gene counts.

### Module 5: Pathway analysis

This module is designed to uncover biological pathways associated with gene expression profiles in the scRNA-seq datasets. By utilizing widely used reference pathway databases and tools, this module facilitates interpretation of cellular processes and signaling mechanisms within clusters, cell types, or custom gene lists. The module integrates *KEGG* (Kyoto Encyclopedia of Genes and Genomes) and *Reactome* to identify relevant pathways using *clusterProfiler* and *ReactomePA*. Users can perform pathway analyses on single or multiple clusters by selecting specific clusters from a dropdown menu. Supported model organisms include human, mouse, and rat, with several adjustable parameters for further customization. It will also generate tables with pathway details and plots similar to those from the GO analysis module.

### Module 6: Gene Set Enrichment Analysis (GSEA) analysis

The GSEA module in ScRDAVis identifies enriched pathways or gene sets based on ranked gene lists, providing insights into pathway-level changes in scRNA-seq datasets. This module leverages curated gene sets from the Molecular Signatures Database (MSigDB) [34] and utilizes the *fgsea* package for analysis. Users can perform GSEA on single or multiple clusters by selecting the desired clusters from a dropdown menu. The output includes a GSEA plot with enrichment scores across ranked gene lists (S2A Fig), highlighting leading-edge subsets. A bar plot summarizes the top enriched pathways ranked by significance (S2B Fig), and the PlotGseaTable combines enrichment scores, p-values, and pathway annotations for detailed interpretation (S2C Fig).

### Module 7: Cell-Cell communication analysis

**Fig 4:**
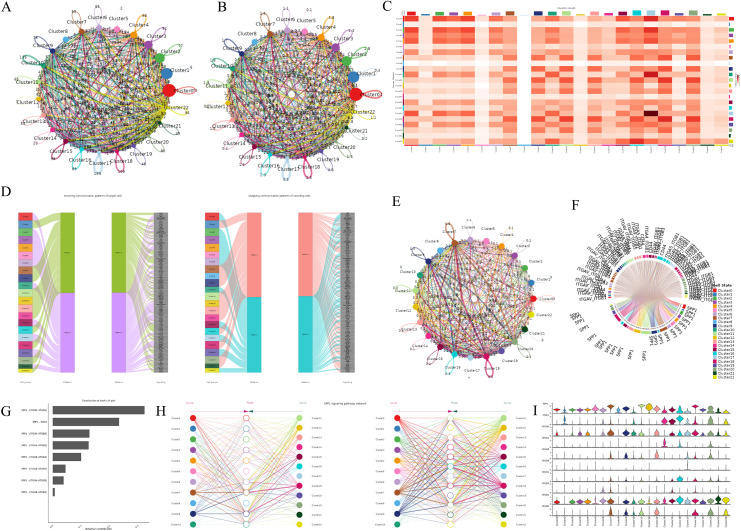
Cell-Cell communication analysis. **A)** Circular plot representing the overall signaling network with directed connections with count and **B)** with weight/strength. **C)** Interaction heatmap showing signaling intensity between cell types or clusters. **D)** Incoming and outgoing cell patterns for clusters. Visualizations of the MHC-II signaling pathway, including **E)** Circle, **F)** Chord, **G)** Bubble, and **H)** Hierarchy plots, along with **I)** Violin plots representing gene expression levels.

This module facilitates the study of signaling interactions between cell types or clusters, uncovering intercellular communication through ligand-receptor interactions. It employs the widely used *CellChat* package to explore signaling pathways and their roles in cellular processes. Adjustable parameters include thresholds for gene expression, log fold-change, p-value, and communication probability for ligand-receptor interactions using methods such as triMean, truncatedMean, or thresholdedMean. Currently, the tool supports human and mouse datasets. Results from the module are presented in multiple visualization formats, including a circular plot representing the overall signaling network with directed connections among the clusters (Fig 4A and 4B), and an interaction heatmap displaying the intensity of interactions between cell types or clusters (Fig 4C). River plots identify patterns of incoming and outgoing signaling (Fig 4D). Users can also select specific signaling pathways to generate similar plots. Here the MHC-II pathway is visualized through circle (Fig 4E), chord (Fig 4F), bubble (Fig 4G), and hierarchy plots (Fig 4H), with gene expression represented by violin plots (Fig 4I).

### Module 8: Trajectory and pseudotime analysis

**Fig 5:**
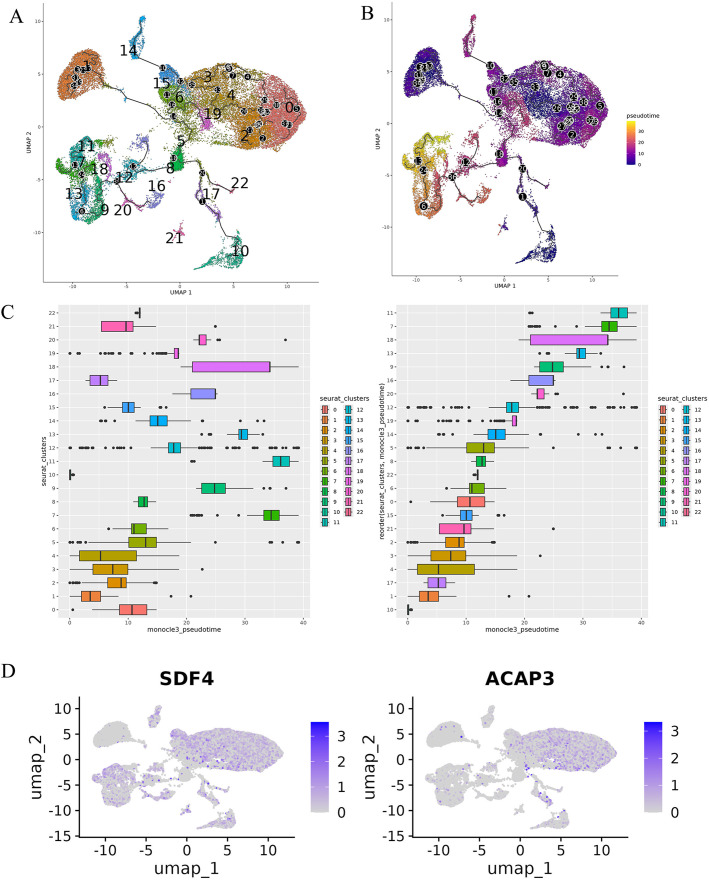
Trajectory and pseudotime analysis. **A)** Trajectory plot showing inferred developmental pathways with annotated branch points, roots, and leaves. **B)** Pseudotime-ordered tables linking cells to pseudotime scores. **C)** Bar plot visualizing pseudotime distributions. **D)** List of dynamically regulated genes identified along pseudotime.

This module employs *Monocle3* to infer developmental trajectories and order cells along pseudotime, revealing dynamic processes and cellular progression in scRNA-seq data. This module allows users to explore changes in gene expression and identify key drivers of cellular differentiation and transitions. Using the GUIs, users can construct developmental trajectories by organizing cells based on their gene expression profiles. The module identifies branch points, roots, and leaves, corresponding to key decision points and endpoints in cellular differentiation, and orders cells along the inferred trajectories to represent their progression through developmental states. Root clusters can be manually selected to define the starting point of pseudotime analysis. Outputs include trajectory plots with annotated branch points (Fig 5A), pseudotime-ordered tables linking cells to pseudotime scores (Fig 5B), bar plots showing pseudotime distributions (Fig 5C), and lists of dynamically regulated genes during pseudotime (Fig 5D).

### Module 9.1: Co-expression network analysis

**Fig 6:**
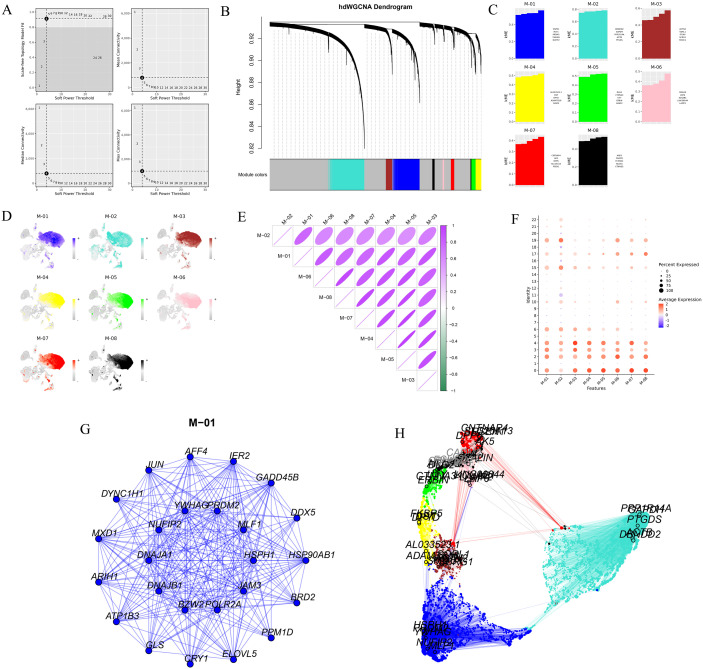
Co-Expression network analysis. **A)** Soft power plot to determine the optimal soft power parameter for network construction. **B)** Module network plot with genes as nodes and edges representing correlations. **C)** Bar plot showing module relationships with experimental conditions. **D)** Feature plot mapping module-specific gene expression across UMAP embeddings. **E)** Correlation matrix between the modules and **F)** Bubble chart illustrating module expression. **G)** hub genes of modules 01, top 10 genes in the middle and the 15 in the outer circle. **H)** UMAP showing co-expressed modules with hub genes.

The Co-expression network analysis module in ScRDAVis leverages the *hdWGCNA* package to identify gene modules and their relationships in scRNA-seq data. This module enables users to construct WGCNA and identify hub genes, transcription factors, and modules associated with specific cellular functions or clusters. Users could select one cluster for analysis, and the module provides outputs such as a soft power plot to determine the optimal soft power parameter for network construction (Fig 6A), a module network plot displaying co-expression networks with genes as nodes and edges representing correlations (Fig 6B), a bar plot highlighting module relationships with experimental conditions (Fig 6C), and feature plots mapping module-specific gene expression across UMAP embeddings (Fig 6D). Additional outputs include a correlation matrix for the modules (Fig 6E), a bubble chart with module expression (Fig 6F), a module network with the top hub genes (Fig 6G), and a UMAP based on the co-expressed modules with the hub genes (Fig 6H).

### Module 9.2: Transcription factor regulatory network analysis

**Fig 7:**
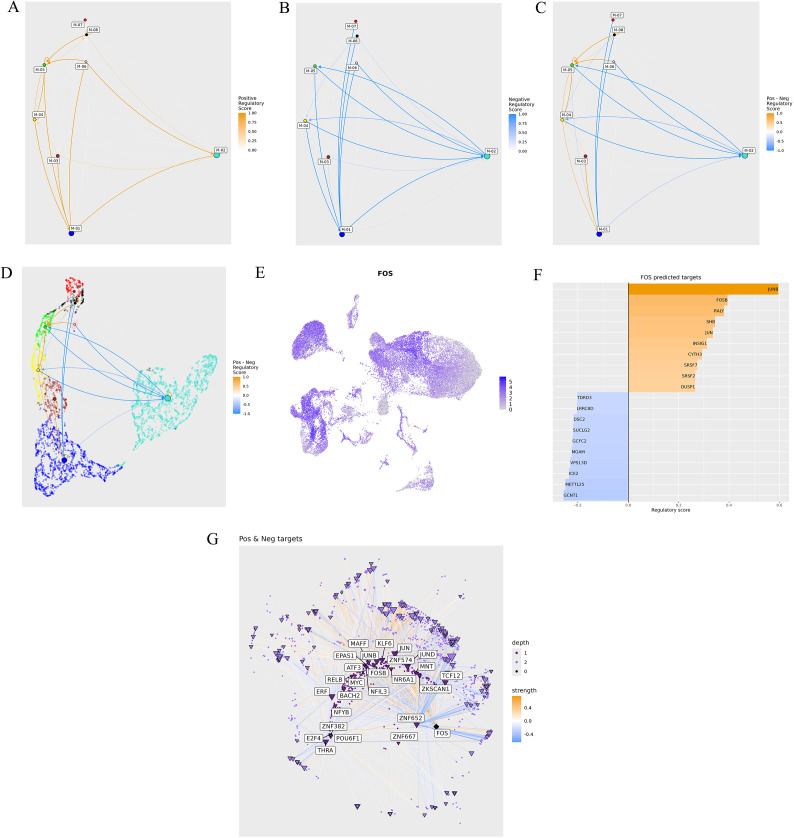
Transcription factor regulatory network analysis. **A)** Module network plots showing interactions between modules with positive and **B**) negative scores. **C)** Combined module network plot. **D)** UMAP-based module network plot with positive and negative scores. **E)** Feature plot for selected transcription factors. **F)** Bar plot highlighting TF contributions across gene modules. **G)** Positive and negative targets for the selected TF.

This module builds on the capabilities of hdWGCNA to identify and analyze TF-mediated regulation within the co-expression networks. It is particularly valuable for uncovering gene regulatory mechanisms driving cellular processes or cluster-specific functions in scRNA-seq data. By utilizing databases like JASPAR 2024 [35], the module maps TFs to their target genes and employs a machine-learning (ML) tool called XGBoost to model TF regulation. Supported model organisms include human and mouse datasets. Outputs include detailed tables and visualizations such as module network plots showing interactions between modules with positive (Fig 7A), negative (Fig 7B), and combined scores (Fig 7C). In addition, the UMAP-based module network plots display positive and negative scores (Fig 7D). After selecting a specific TF from the dropdown menu, users can generate feature plots for the TF (Fig 7E), bar plots highlighting TF contributions across the gene modules (Fig 7F), and positive and negative targets for a selected TF (Fig 7G).

### Case study: Application of ScRDAVis to GSE266873

To demonstrate the utility and comprehensiveness of ScRDAVis, we applied the tool to a publicly available dataset (GSE266873) from the NCBI’s GEO resource [23]. This dataset profiles the immune landscape during perihematomal edema progression after intracerebral hemorrhage (ICH) using single-cell RNA sequencing. It includes 9 samples across three temporal groups: Group 1 (G1: 0–6 hours post-ICH, n = 3; GSM8255340–GSM8255342), Group 2 (G2: 6–24 hours post-ICH, n = 3; GSM8255343–GSM8255345), and Group 3 (G3: 24–48 hours post-ICH, n = 3; GSM8255346–GSM8255348). Users can directly download all raw matrices, features, and barcodes as a compressed tar archive file (https://www.ncbi.nlm.nih.gov/geo/download/?acc=GSE266873&format=file), extract the files locally, and upload the combined 27 files into ScRDAVis to initiate the workflow. A step-by-step approach for analyzing this dataset, along with the parameters used at each stage, is fully documented in the Manual tab of ScRDAVis.

#### Analysis workflow with ScRDAVis.

The ScRDAVis pipeline begins with Module 1, which supports single- or multi-sample analysis. After preprocessing, QC filtering and normalization, PCA was performed with default 50 PCs, with refinement guided by the JackStraw procedure implemented within the Seurat tab. Clustering then identifies distinct immune cell populations, which can be visualized using UMAP/t-SNE, followed by marker gene identification and cell-type annotation with ScType, SingleR, GPTCelltype, or manual curation. Condition-based differential expression analysis further reveals transcriptional programs distinguishing Group1, Group2, and Group3 (Fig 2). Subsequent modules enable deeper exploration of these groups of cells as follows. Subclustering (Module 2) allows refinement of specific populations. Correlation network analysis (Module 3) (S1 Fig) highlights pairwise similarities across cell clusters, while GO (Module 4) (Fig 3) and pathway analysis (Module 5) identifies enriched biological processes and signaling cascades during edema progression. GSEA (Module 6) performs pathway enrichment using ranked differential expression results (S2 Fig). Cell–cell communication analysis (Module 7) uncovers evolving immune signaling networks, including MHC-II–mediated interactions, visualized through circular, chord, bubble, and hierarchy plots (Fig 4). Trajectory and pseudotime analysis (Module 8) helps reconstruct cellular state transitions across post-ICH timepoints (Fig 5). Finally, network-based modules provide mechanistic insights that include co-expression networks (Module 9.1) revealing hub genes and module–condition relationships (Fig 6), while transcription factor regulatory networks (Module 9.2) identifies candidate TFs orchestrating immune responses during edema progression (Fig 7). This case study demonstrates how ScRDAVis enables an end-to-end exploration of scRNA-seq data, from QC and clustering to high-level functional interpretation and regulatory inference (Fig 1, workflow overview). By integrating nine analytical modules in a browser-based framework, ScRDAVis empowers researchers to systematically dissect cellular heterogeneity, cellular dynamics, and regulatory programs across complex conditions such as ICH. Importantly, the modular design, adjustable parameters, and the built-in documentation ensure both reproducibility and flexibility.

## Discussion

ScRDAVis is a comprehensive and user-friendly toolkit designed to streamline scRNA-seq analysis workflows specifically designed for biologists with little or no programming knowledge. It incorporates a suite of nine seamlessly connected modules that enable researchers to address key challenges with the current tools used for analyzing single-cell transcriptomic datasets. Table 1 highlights that while current GUI-based tools facilitate basic exploratory analyses, they lack comprehensive functionalities such as trajectory analysis, pathway enrichment, and cell-cell communication features. ScRDAVis addresses these limitations by integrating state-of-the-art methodologies and widely used bioinformatics tools, bridges the gap between complex computational functionality and intuitive usability through an accessible, user-friendly R Shiny interface. One of the notable strengths of ScRDAVis is its flexibility in handling single or multiple datasets, making it suitable for analyzing data from diverse experimental designs to address complex biological questions. Modules like single or multiple sample analysis and subclustering empower researchers to explore both the broad and granular aspects of cellular heterogeneity, providing insights into cell population-level trends and subpopulation-specific dynamics. The seamless integration of QC filtering, normalization, dimensionality reduction, and clustering ensures robust and reproducible results. ScRDAVis also excels in visualizing data through a variety of customizable plots, including UMAP, t-SNE, dot plots, and heatmaps, which enable clear and interpretable representation of complex biological patterns. This functionality is complemented by its capacity for cell type annotation by leveraging tools such as *ScType*, *SingleR*, and AI-driven *GPTCelltype* to enhance cell classification accuracy.

Modules like correlation network analysis and pathway analysis extend the scope of single-cell investigations by uncovering gene-gene interactions and biological pathways underpinning cellular functions. The incorporation of KEGG, Reactome, and Gene Ontology databases allows for contextualizing their findings within the established biological frameworks; thus, enhancing the interpretability of results. The trajectory and pseudotime analysis module provide a detailed view of cellular differentiation and lineage progression, offering insights into developmental processes and key regulatory genes. Additionally, the cell-cell communication module elucidates intercellular signaling through ligand-receptor interactions, highlighting the roles of signaling pathways. The inclusion of Co-expression network analysis and TF regulatory network analysis adds further depth to the toolkit. These modules enable users to identify co-expressing modules, hub genes, and TFs driving specific cellular states or responses. By integrating data from databases like JASPAR and employing ML techniques such as XGBoost, ScRDAVis provides a powerful framework for unraveling transcriptional regulation. ScRDAVis emphasis on modularity and interconnectivity allows users to transition seamlessly between a rich set of diverse analyses. For instance, results from subclustering can be fed into pathway or trajectory analyses, while co-expression networks can inform TF studies. This interconnected approach not only reduces the complexity of scRNA-seq workflows but also fosters holistic biological interpretations. Despite its strengths, ScRDAVis does have certain limitations. The reliance on predefined databases may pose challenges for researchers studying less characterized organisms or conditions. Additionally, while the toolkit supports human and mouse datasets extensively, enhancing compatibility to other model organisms could broaden its applicability.

## Availability and future directions

The standalone application code can be downloaded at https://github.com/GudaLab/ScRDAVis. The online version of ScRDAVis is accessible at https://www.gudalab-rtools.net/ScRDAVis.

ScRDAVis represents a significant advancement in single-cell transcriptomics, combining robust analytical capabilities with intuitive visualization and interpretation tools. Its modular design, broad functionality, and integration of cutting-edge tools make it a valuable resource for researchers aiming to unlock the complexity of cellular heterogeneity and cell dynamics in scRNA-seq datasets. As single-cell technologies continue to evolve, ScRDAVis provides a solid foundation for tackling the challenges of modern biological research. In the future, we aim to improve ScRDAVis by incorporating more third-party tools for upstream and downstream analyses and user-friendly features based on the user feedback.

## Supporting information

S1 Table List of packages used in ScRDAVis. (DOCX)

S2 Table Description of various parameters used in ScRDAVis. This supplementary table summarizes all the adjustable parameters available in the ScRDAVis web application. Each parameter is linked to specific analytical modules, including marker identification, cell type prediction, plot customization, correlation and enrichment analysis, trajectory inference, co-expression modules, and transcription factor regulatory networks. Default values and brief descriptions of their function are included to support reproducibility and interpretation of the analysis workflows.
(DOCX)

S3 Table Estimated runtime for analysis of different tasks in ScRDAVis. (DOCX)

S1 Fig Correlation network analysis. A) Heatmap depicting pairwise correlation values between the cell clusters. B) Correlation network plot between the cell clusters.
(TIF)

S2 Fig GSEA analysis. A) GSEA plot showing enrichment scores across ranked gene lists, highlighting leading-edge subsets. B) Bar plot summarizing the top enriched pathways ranked by significance. C) Table plot (PlotGseaTable) combining enrichment scores, p-values, and pathway annotations for detailed interpretation.
(TIF)
