# Supplementary material for: ScRDAVis: An R shiny application for single-cell transcriptome data analysis and visualization
Source: PLoS Comput Biol. 2025 Nov 13;21(11):e1013721. doi: 10.1371/journal.pcbi.1013721 (PMC12626302; doi:10.1371/journal.pcbi.1013721)
Supplement: S1 Table — (DOCX) [file pcbi.1013721.s001.docx]

**S1 Table**. List of packages used in ScRDAVis

| Packages | Usage | Link |
| --- | --- | --- |
| **Web Applications and Interactivity** | | |
| shiny | Building interactive web applications | <https://github.com/rstudio/shiny> |
| shinythemes | Provides themes for shiny apps | <https://github.com/rstudio/shinythemes> |
| shinyjs | Enhances shiny apps with JavaScript | <https://github.com/daattali/shinyjs> |
| shinyFiles | File management in shiny | <https://github.com/thomasp85/shinyFiles> |
| shinyWidgets | Additional widgets for shiny | <https://dreamrs.github.io/shinyWidgets> |
| shinycssloaders | Loaders and spinners for shiny apps | <https://github.com/daattali/shinycssloaders> |
| shinydashboard | Dashboards for shiny applications | <https://github.com/rstudio/shinydashboard> |
| DT | Interactive data tables in shiny | <https://github.com/rstudio/DT> |
| **Data Manipulation and Visualization** | | |
| ggplot2 | Data visualization based on the Grammar of Graphics | <https://ggplot2.tidyverse.org> |
| data.table | High-performance data manipulation | <https://github.com/Rdatatable/data.table> |
| dplyr | Data manipulation | <https://dplyr.tidyverse.org> |
| tibble | Data frame-like structure | <https://tibble.tidyverse.org> |
| ggpubr | Publication-ready plots | <https://rpkgs.datanovia.com/ggpubr> |
| gridExtra | Arranging multiple plots | <https://cran.r-project.org/web/packages/gridExtra/index.html> |
| ggalluvial | Alluvial diagrams for categorical data | <https://cran.r-project.org/web/packages/ggalluvial/vignettes/ggalluvial.html> |
| ggrepel | Better label placement in ggplot2 | <https://ggrepel.slowkow.com> |
| cowplot | Streamlined plot themes | <https://github.com/wilkelab/cowplot> |
| ggupset | Upset plots in ggplot2 | <https://github.com/const-ae/ggupset> |
| circlize | Circular visualizations, such as chord diagrams and circular heatmaps | <https://github.com/jokergoo/circlize> |
| hdf5r | Reading and writing HDF5 data, commonly used for large datasets | <https://github.com/hhoeflin/hdf5r> |
| pdftools | PDF processing and text extraction | <https://github.com/ropensci/pdftools> |
| R.utils | Various programming utilities | <https://github.com/HenrikBengtsson/R.utils> |
| openxlsx | Excel file handling | <https://github.com/ycphs/openxlsx> |
| patchwork | Combining ggplots | <https://github.com/thomasp85/patchwork> |
| **Statistical Analysis and Machine Learning** | | |
| openai | Interface for using OpenAI's APIs, including GPT models | <https://github.com/irudnyts/openai> |
| metap | Meta-analysis | <https://cran.r-project.org/web/packages/metap/index.html> |
| xgboost | Extreme Gradient Boosting | <https://github.com/dmlc/xgboost> |
| NMF | Non-negative matrix factorization | [http://renozao.github.io/NMF](http://renozao.github.io/NMF/) |
| glmGamPoi | GLM-based overdispersion models | <https://github.com/const-ae/glmGamPoi> |
| presto | Fast methods for statistical analysis, often in genomics | <https://github.com/immunogenomics/presto> |
| **Bioinformatics and Genomics** | | |
| BiocManager | Manages BioConductor packages | <https://www.bioconductor.org/install> |
| Seurat  SeuratObject  SeuratWrappers  SeuratDisk  Sctransform  scRNAseq | Single-cell RNA sequencing analysis | <https://github.com/satijalab/seurat>  <https://github.com/satijalab/seurat-object>  <https://github.com/satijalab/seurat-wrappers>  <https://github.com/mojaveazure/seurat-disk>  <https://github.com/satijalab/sctransform>  [https://bioconductor.org/packages/scRNAseq](https://bioconductor.org/packages/scRNAseq/) |
| DoubletFinder | To detect doublets | <https://github.com/chris-mcginnis-ucsf/DoubletFinder> |
| scran | Single-cell RNA-seq analysis and normalization | [https://github.com/MarioniLab/scran](https://github.com/MarioniLab/scran/) |
| harmony | Integration and batch effect correction, often in single-cell genomics | <https://github.com/immunogenomics/harmony> |
| celldex  SingleR  GPTCelltype  ScType | Cell annotation and RNA-seq analysis | [https://bioconductor.org/packages/celldex](https://bioconductor.org/packages/celldex/)  [https://bioconductor.org/packages/SingleR](https://bioconductor.org/packages/SingleR/)  <https://github.com/Winnie09/GPTCelltype>  <https://github.com/IanevskiAleksandr/sc-type> |
| WGCNA  hdWGCNA | Weighted gene co-expression network analysis | <https://cran.r-project.org/web/packages/WGCNA/index.html>  <https://smorabit.github.io/hdWGCNA> |
| Motifmatchr  TFBSTools  JASPAR2024 | Motif analysis and transcription factor binding sites | [https://bioconductor.org/packages/motifmatchr](https://bioconductor.org/packages/motifmatchr/)  [https://bioconductor.org/packages/TFBSTools](https://bioconductor.org/packages/TFBSTools/)  <https://bioconductor.org/packages/JASPAR2024> |
| CellChat | Cell-cell communication analysis | <https://github.com/sqjin/CellChat> |
| clusterProfiler  ReactomePA  Enrichplot  enrichR | Functional enrichment analysis | [https://bioconductor.org/packages/clusterProfiler](https://bioconductor.org/packages/clusterProfiler/)  [https://bioconductor.org/packages/ReactomePA](https://bioconductor.org/packages/ReactomePA/)  [https://bioconductor.org/packages/enrichplot](https://bioconductor.org/packages/enrichplot/)  <https://www.rdocumentation.org/packages/enrichR/versions/3.2> |
| msigdbr  msigdbdf  fgsea | Access to the MSigDB gene sets and Fast Gene Set Enrichment Analysis | [https://igordot.github.io/msigdbr](https://igordot.github.io/msigdbr/)  <https://github.com/ToledoEM/msigdf>  [https://bioconductor.org/packages/fgsea](https://bioconductor.org/packages/fgsea/) |
| ComplexHeatmap | Complex heatmaps | [https://bioconductor.org/packages/ComplexHeatmap](https://bioconductor.org/packages/ComplexHeatmap/) |
| HGNChelper | Handling gene symbols | <https://github.com/waldronlab/HGNChelper> |
| org.Hs.eg.db  org.Mm.eg.db  org.Mmu.eg.db  org.Rn.eg.db  org.Ss.eg.db | Organism-specific annotation databases | [https://bioconductor.org/packages/org.Hs.eg.db](https://bioconductor.org/packages/org.Hs.eg.db/)  [https://bioconductor.org/packages/org.Mm.eg.db](https://bioconductor.org/packages/org.Mm.eg.db/)  [https://bioconductor.org/packages/org.Mmu.eg.db](https://www.bioconductor.org/packages/org.Mmu.eg.db/)  [https://bioconductor.org/packages/org.Rn.eg.db](https://www.bioconductor.org/packages/org.Rn.eg.db/)  [https://bioconductor.org/packages/org.Ss.eg.db](https://bioconductor.org/packages/org.Ss.eg.db/) |
| GenomicRanges  EnsDb.Hsapiens.v86  EnsDb.Mmusculus.v79 | Genomic data representation | <https://bioconductor.org/packages/GenomicRanges>  [https://bioconductor.org/packages/EnsDb.Hsapiens.v86](https://bioconductor.org/packages/EnsDb.Hsapiens.v86/)  [https://bioconductor.org/packages/EnsDb.Mmusculus.v79](https://bioconductor.org/packages/EnsDb.Mmusculus.v79/) |
| BSgenome.Hsapiens.UCSC.hg38  BSgenome.Mmusculus.UCSC.mm10 | Genomic sequences | [https://bioconductor.org/packages/BSgenome.Hsapiens.UCSC.hg38](https://www.bioconductor.org/packages/BSgenome.Hsapiens.UCSC.hg38/)  [https://bioconductor.org/packages/BSgenome.Mmusculus.UCSC.mm10](https://bioconductor.org/packages/BSgenome.Mmusculus.UCSC.mm10/) |
| EnhancedVolcano | Volcano plots for differential expression analysis | <https://github.com/kevinblighe/EnhancedVolcano> |
| multtest | Multiple hypothesis testing | [https://bioconductor.org/packages/multtest](https://www.bioconductor.org/packages/multtest/) |
| genesorteR | Gene sorting for specific analysis | <https://github.com/mahmoudibrahim/genesorteR> |
| **Network and Graph Analysis** | | |
| igraph | Network analysis | <https://github.com/igraph/rigraph> |
| ggraph | Visualization of graph networks | <https://github.com/thomasp85/ggraph> |
