# Supplementary material for: ScRDAVis: An R shiny application for single-cell transcriptome data analysis and visualization
Source: PLoS Comput Biol. 2025 Nov 13;21(11):e1013721. doi: 10.1371/journal.pcbi.1013721 (PMC12626302; doi:10.1371/journal.pcbi.1013721)
Supplement: S2 Table — This supplementary table summarizes all the adjustable parameters available in the ScRDAVis web application. Each parameter is linked to specific analytical modules, including marker identification, cell type prediction, plot customization, correlation and enrichment analysis, trajectory inference, co-expression modules, and transcription factor regulatory networks. Default values and brief descriptions of their function are included to support reproducibility and interpretation of the analysis workflows. (DOCX) [file pcbi.1013721.s002.docx]

**S2 Table**. Description of various parameters used in ScRDAVis

This supplementary table summarizes all adjustable parameters available in the ScRDAVis web application. Each parameter is linked to specific analytical modules, including marker identification, cell type prediction, plot customization, correlation and enrichment analysis, trajectory inference, co-expression modules, and transcription factor regulatory networks. Default values and brief descriptions of their function are included to support reproducibility and interpretation of the analysis workflows.

| **Parameter Name** | **Default Value** | **Description** |
| --- | --- | --- |
| **File upload and Stats** | | |
| Upload H5 File (Cell Ranger Output) | H5 | Rename it to SAMPLE_NAME.h5 for proper identification. |
| Upload Matrix files (mtx, features, barcodes) | Matrix | Rename as SAMPLE_NAME_matrix.mtx.gz, SAMPLE_NAME_features.tsv.gz, and SAMPLE_NAME_barcodes.tsv.gz, and upload together as a set. |
| Upload Seurat Object | RDS | Filename.rds (Seurat object). The orig.ident attribute should match the sample name(s). |
| Upload Matrix Count File (.txt) | TXT | Filename.txt with rows as genes and columns as sample_cellID. |
| Minimum cell expression per gene | 0 | Minimum number of cells that must express a given gene to retain it. |
| Minimum gene expression per cell | 0 | Minimum number of genes that must be expressed in a cell to retain it. |
| **Sample Groups and QC Filtering** | | |
| Number of groups | 1 to 6 | Select up to 6 groups. |
| Group 1 Name | Group1 | Type the group name. |
| Group 2 Name | Group2 | Type the group name. |
| Group 3 Name | Group3 | Type the group name. |
| Group 4 Name | Group4 | Type the group name. |
| Group 5 Name | Group5 | Type the group name. |
| Group 6 Name | Group6 | Type the group name. |
| Min gene count per cell | 0 | Filters out cells with fewer than this number of genes expressed.[Recommended: 200 to 500] |
| Max gene count per cell | 7500 | Filters out cells with more than this number of genes expressed. [Recommended: 5000 to 7500] |
| Max mitochondrial % | 5 | Removes cells with excessive mitochondrial gene expression, often indicating low-quality or dying cells. [Recommended: <10%]. |
| **Normalization and PCA Analysis** | | |
| Scale factor | 10000 | Scale factor used in LogNormalize method for total expression normalization. Min: 1, Max: 1e6 |
| Variable gene method | vst | Method for selecting variable features: vst (default), mean.var.plot, or dispersion. |
| Number of variable genes | 2000 | Number of top variable genes to retain for downstream analysis. Min: 100, Max: 10000 |
| PCA dimensions | 50 | Number of principal components computed for dimensionality reduction. Min: 2, Max: 100 |
| JackStraw max dims | 20 | Maximum number of PCs tested for significance in JackStraw analysis. Min: 1, Max: 100 |
| JackStraw num.replicate | 100 | Number of permutations used in JackStraw resampling. Min: 1, Max: 1000 |
| JackStraw plot max PCs | 20 | Maximum PCs to display in JackStraw significance plot. Min: 10, Max: 100 |
| SCTransform | Negative binomial regression | Uses regularized negative binomial regression for clustering and differential expression |
| **Clustering** | | |
| Clustering resolution | 0.5 | Resolution used for cluster granularity. Higher = more clusters. Min: 0.1, Max: 1 |
| Clustering algorithm | Leiden | Graph-based clustering algorithm: Louvain (1), SLM (3), or Leiden (4). |
| Integration method | Nones | Data integration method. If 'None', no integration is performed. |
| HarmonyIntegration | Reduction = harmony; Distance = Cosine | Batch correction using Harmony with cosine distance. |
| CCAIntegration | Reduction = cca; Distance = Euclidean | Canonical correlation analysis for dataset integration. |
| RPCAIntegration | Reduction = rpca; Distance = Euclidean | Faster, scalable variant of CCA. |
| JointPCAIntegration | Reduction = jointpca; Distance = Euclidean | Joint PCA embedding for multi-dataset integration. |
| UMAP k-nearest-neighbours | 20 | Number of nearest neighbors considered for UMAP. Min: 2, Max: 50 |
| UMAP dims | 30 | Number of PCs used for UMAP dimensionality reduction. Min: 2, Max: 100 |
| UMAP min.dist | 0.3 | Controls how tightly UMAP clusters points. Smaller = more tightly packed. Min: 0.001, Max: 0.5 |
| tSNE dims | 30 | Number of PCs used for t-SNE dimensionality reduction. Min: 2, Max: 100 |
| **Remove Doublets** | | |
| Doublet rate | 0.075 | Expected proportion of doublets in the sample. Min: 0.075, Max: 0.1 |
| keep or remove doublets | Remove doublets | To keep or remove doublets |
| **Marker Identification** | | |
| FindAllMarkers | Select all cluster | Identifies marker genes for each cluster compared to all other cells. |
| FindMarkers | Select one cluster against another cluster | Finds differentially expressed genes between two specific groups of cells. |
| FindConservedMarkers | Select one cluster to check for conserved in all clusters | Identifies markers that are conserved across multiple groups (e.g., conditions or batches). |
| min.pct | 0.25 | Minimum fraction of cells expressing the gene for it to be tested. Min: 0.01, Max: 1.0 |
| logfc.threshold | 0.25 | Minimum log fold change required to consider gene differentially expressed. Min: 0.01, Max: ∞ |
| Statistical test | wilcox | Statistical test used for differentially expressed gene or marker identification. |
| Return only positive markers | Yes | Whether to return only genes upregulated in the target group. |
| **Cell Type Prediction** | | |
| Cell type method | SingleR | Methods for cell type prediction. ScType, SingleR, GPTCelltype, Use Own Labels |
| Reference tissue for SingleR | hpca | Reference data sources for SingleR annotation. hpca, blueprint_encode, mouse_rnaseq, immgen, dice, novershtern_hematopoietic, monaco_immune |
| DE method for SingleR | classic | SingleR Differential expression method used for prediction scoring. (classi, wilcox, t test). |
| Reference data for ScType | Immune system | Selected cell type reference for matching. |
| Top genes prediction GPTCelltype | 10 | Number of top genes used for GPTCelltype or other predictions. |
| Model for GPTCelltype | gpt-5 | OpenAI models available in GPTCelltype. gpt-5, gpt-5-mini, gpt-5-nano, gpt-4, gpt-4o, gpt-4-turbo, gpt-3.5-turbo, etc. |
| Use Own Labels | Cluster 0 to Cluster N | This option allows users to manually assign custom names to clusters. Users may enter identical names for two or more clusters if they wish to merge them into a single group |
| **Cluster-Based Plots** | | |
| No. of features to display | 3 | Number of genes to visualize per plot or select the list of gene names from the dropdown and type the specific genes which you are interested in eg: KLk2,KLK3,CTSG,MS4A3. |
| Select one or multiple cluster(s) for plotting | Default all clusters | User can adjust the cluster to plot. |
| Plot type | Dot Plot | Types of visualizations for gene expression or differentially expressed genes. (Dot Plot, Violin Plot, Ridge Plot, Feature Plot, Volcano Plot). |
| Dim plot labels | No | Whether to display labels in dimensionality reduction plots. |
| Group.by | condition | Grouping variable for DE or plotting, e.g., condition or sample ID. |
| Split.by | NULL | Whether to split plots by condition, sample, or not at all. |
| **Condition Based Plots** | | |
| Select the Condition1 | Group1 | User can select any one condition. |
| Select the Condition2 | Group2 | User can select any one condition. |
| min.pct | 0.25 | Minimum fraction of cells expressing the gene to be tested in marker analysis. Min: 0.01, Max: 1.0 |
| logfc.threshold | 0.25 | Log fold change threshold for identifying differentially expressed genes. Min: 0.01, Max: ∞ |
| Statistical test | wilcox | Test used for differential expression: e.g., wilcox, wilcox_limma, bimod, roc, t, LR, MAST. |
| Positive markers only | Yes | If Yes, return only genes upregulated in the target group. |
| group.by | condition | Metadata variable to group cells during marker analysis. (Condition and samples). |
| Plot type | Dot Plot | Types of visualizations for gene expression or differentially expressed genes. (Dot Plot, Violin Plot, Ridge Plot, Feature Plot, Volcano Plot. |
| Number of features to display | 3 | Number of genes to visualize per plot or select the list of gene names from the dropdown and type the specific genes which you are interested in eg: KLk2,KLK3,CTSG,MS4A3 |
| **Subclustering** | | |
| Cluster Type Selection | Seurat clusters | Choose source for subclustering (Seurat, predicted, or gene-based selection. |
| Select cluster(s) | Select the cluster default 0 | Generated based on selected cluster type. |
| Genes to include (positive selection) | Eg: FCN1 or FCN1,PSAP | Enter comma-separated gene symbols for subsetting. |
| Genes to exclude (negative selection) | Eg: FCN1 or FCN1,PSAP | Enter comma-separated gene symbols to exclude cells. |
| **Correlation** | | |
| Input data | Output of single or multiple samples | Select the input from full dataset or subclustering. |
| Celltype method | Seurat clusters | Select celltype grouping for correlation. (Seurat clusters or predicted). |
| Correlation method | Spearman | Method to compute correlation between clusters (Pearson, Spearman, Kendall). |
| **Gene Ontology** | | |
| Input data | Output of single or multiple samples | Choose the source of genes for GO analysis. |
| Celltype method | Seurat clusters | Clustering source for gene selection. (Seurat clusters or predicted). |
| Organism | Human | Organism-specific annotation package. (Human, Mouse, Rat, Pig, Rhesus). |
| Ontology | BP | GO ontology cate.g.ories: biological process, etc. (BP, MF, CC, ALL). |
| pAdjustMethod | BH | Method for p-value adjustment. (holm, bonferroni, BH, BY, fdr, none). |
| pvalueCutoff | 0.05 | Significance threshold for raw p-value. Min: 0, Max: 1 |
| qvalueCutoff | 0.2 | Significance threshold for q-value. Min: 0, Max: 1 |
| Minimal gene size | 10 | Minimum number of genes in a category. Min: 1, Max: 500 |
| Maximal gene size | 500 | Maximum number of genes in a category. Min: 10, Max: 5000 |
| Plot type | Dotplot | Visualization options for enriched GO terms. (dotplot, barplot, cnetplot, upsetplot). |
| Top categories to plot | 10 | Number of categories to include in plots. Min: 1, Max: 50 |
| **Pathway Analysis** | | |
| Pathway analysis type | KEGG | Source of pathway database. (KEGG or Reactome). |
| Input data | Output of single or multiple samples | Choose the source of genes for pathway analysis. |
| Celltype method | Seurat clusters | Clustering source for gene selection. (Seurat clusters or predicted). |
| Organism | Human | Organism-specific annotation package. (Human, Mouse, Rat). |
| pAdjustMethod | BH | Adjustment for multiple testing. (holm, bonferroni, BH, BY, fdr, none). |
| pvalueCutoff | 0.05 | Significance threshold for raw p-value. Min: 0, Max: 1 |
| qvalueCutoff | 0.2 | Significance threshold for q-value. Min: 0, Max: 1 |
| Minimal gene size | 10 | Minimum number of genes in pathway. Min: 5, Max: 500 |
| Maximal gene size | 500 | Maximum number of genes in pathway. Min: 15, Max: 5000 |
| Plot type | Dotplot | Type of plot for pathway enrichment. (dotplot, barplot, cnetplot, upsetplot). |
| Top categories to plot (Pathway) | 10 | Number of enriched pathways shown. Min: 1, Max: 50 |
| **GSEA Analysis** | | |
| Input data | Output of single or multiple samples | Choose the source of genes for GSEA analysis. |
| Celltype method | Seurat clusters | Clustering source for gene selection. (Seurat clusters or predicted). |
| Organism | Homo sapiens | Species-specific gene set database. (Homo sapiens, Mus musculus). |
| MSigDB category | Hallmark gene sets (H) | Gene set collection from MSigDB. (H, C1, C2, C3, C4, C5, C6, C7, C8). |
| ScoreType | std | Controls whether to score all, positive or negative enrichment. (std, pos, neg.). |
| Minimal gene size | 15 | Minimum genes per gene set. Min: 5, Max: 500 |
| Maximal gene size | 50 | Maximum genes per gene set. Min: 15, Max: 5000 |
| Permutations | 100 | Number of random permutations to compute significance. Min: 10, Max: 10000 |
| Plot type | GSEA plot | Style of plot for GSEA results. (GSEA plot, plotGseaTable, barplot). |
| Top significant results to plot | 10 | Number of enriched gene sets to plot. Min: 1, Max: 50 |
| **Cell-Cell Communication Analysis (Cell-chat)** | | |
| Input data | Output of single or multiple samples | Source of expression data for CellChat. |
| Celltype method | Seurat clusters | Cell grouping used in communication analysis. (Seurat clusters or predicted). |
| Organism | PPI.human | Organism-specific protein-protein interaction database. (PPI.human, PPI.mouse). |
| Min % cells expressed | 0 | Minimum percent of cells expressing ligand/receptor. Min: 0, Max: 100 |
| LogFC threshold | 0 | Minimum log fold change for expression filter. Min: 0, Max: 10 |
| P-value threshold | 0.05 | Significance cutoff for ligand-receptor pairs. Min: 0.0001, Max: 1 |
| Averaging method | triMean | Method for averaging gene expression per group. (triMean, truncatedMean, thresholdedMean, median). |
| Minimum cell count | 10 | Minimum number of cells in a group. Min: 5, Max: 1000 |
| Pattern k-value | 2 | Number of communication patterns to infer. Min: 2, Max: 20 |
| Show label | Yes | Display labels on communication plots. |
| Specific Signaling Pathways | The default 1st one is selected | Display the communication for the selected. |
| **Trajectory & Pseudotime Analysis (Monocle3)** | | |
| Input data | Output of single or multiple samples | Select the processed input source. |
| Celltype method | Seurat clusters | Grouping variable for pseudotime. |
| use_partition | No | Whether to use partitioned cell sets. |
| close_loop | Yes | Allow trajectory graph to close loops. |
| label_groups_by_cluster | No | Whether to show cluster labels. |
| label_branch_points | Yes | Show pseudotime branch points. |
| label_roots | Yes | Show root cells in trajectory. |
| label_leaves | No | Show leaf cells in trajectory. |
| Order cell in Pseudotime | Select one cluster as the root | Displays all clusters. |
| Gene functional change (neighbor_graph) | principal_graph | Graph type for trajectory inference. principal_graph, knn |
| Top genes to display in feature plot | 5 | Number or list of genes to plot along pseudotime. |
| **Co-expression & Network Analysis (hdWGCNA)** | | |
| Input data | Output of single or multiple samples | Select the processed input source. |
| Celltype method | Seurat clusters | Seurat cluster or predicted. |
| Select any one cluster | Default 0 | List all the clusters or names. |
| Input data | Output of single or multiple samples | Select the processed input source. |
| Reduction type | UMAP | Dimensionality reduction for module visualization. (UMAP or PCA). |
| Select soft-power Network type | signed | Type of WGCNA correlation network. (signed, unsigned, signed hybrid). |
| Module eigengenes and connectivity Scale model | linear | Statistical model for eigengene computation. (linear, poisson, negbinom). |
| Harmonized eigengenes | Yes | Whether to harmonize eigengenes across datasets. |
| Nearest neighbors (k) | 10 | K for building metacells. Min: 1, Max: 100 |
| Minimum cell group size | 10 | Minimum cells in a group to build a metacell. Min: 5, Max: 100 |
| Max shared cells | 15 | Max overlap between metacells. Min: 1, Max: 100 |
| Target metacells | 1000 | Max number of metacells to construct. Min: 50, Max: 5000 |
| Hub genes per module | 5 | Number of top hub genes labeled. Min: 1, Max: 50 |
| Show inter-module edges | No | Whether to draw edges across modules. |
| **Transcription Factor Regulatory Network Analysis (hdWGCNA)** | | |
| Organism | Human | Reference genome annotation. (Human or Mouse). |
| XGBoost max_depth | 1 | Tree depth for motif-based TF prediction. Min: 1, Max: 10 |
| eta | 0.1 | Learning rate in XGBoost. Min: 0.01, Max: 1 |
| alpha | 0.5 | Regularization parameter. Min: 0, Max: 1 |
| Regulatory score threshold | 0.01 | Minimum score for defining TF-gene edge. Min: 0, Max: 1 |
| Top TFs per gene | 10 | Top regulators retained per gene. Min: 1, Max: 50 |
| Positive regulon threshold | 0.05 | Minimum expression for positive regulons. Min: 0, Max: 1 |
| Negative regulon threshold | -0.05 | Threshold for defining negative regulons. Min: -1, Max: 0 |
| Color network edge by | Cor | TF network edge attribute. (Cor, Gain). |
| Extend TF network layers | Primary and secondary | Depth of TF-target extension. (Primary or Primary and secondary or Primary, secondary and tertiary). |
