# Supplementary material for: ScRDAVis: An R shiny application for single-cell transcriptome data analysis and visualization
Source: PLoS Comput Biol. 2025 Nov 13;21(11):e1013721. doi: 10.1371/journal.pcbi.1013721 (PMC12626302; doi:10.1371/journal.pcbi.1013721)
Supplement: S3 Table — (DOCX) [file pcbi.1013721.s003.docx]

**S3 Table.** Estimated runtime for analysis of different tasks in ScRDAVis.

| **Tab Name** | **Estimated Time** | **Notes** |
| --- | --- | --- |
| Stats | 1–2 minutes | Upload & initial QC plots. H5 is faster; RDS or raw matrix takes longer. |
| Sample Groups & QC Filtering | 1–2 minutes | Depends on number of samples and filtering thresholds. |
| Normalization & PCA | 2–5 minutes | SCTransform takes longer than LogNormalize. |
| JackStraw Analysis | 10–30 minutes | Depends on number of PCs (e.g., 20–50) and resampling (e.g., 100 reps). |
| Clustering & UMAP/tSNE | 1–3 minutes | Slightly longer for large datasets or high resolution. |
| Doublet Detection | 3–30 minutes | Depends on dataset size and expected doublet rate. |
| Marker Identification | 1–3 minutes | Multiple clusters increase runtime (e.g., 10+ clusters). |
| Cell Type Prediction | 2–15 minutes | ScType & SingleR are fast; GPTCelltype depends on OpenAI API latency. |
| Cluster-Based Plots | <1 minute | Faster for fewer genes and features. |
| Condition-Based DEG Analysis | 1–2 minutes | Similar to marker detection; volcano plot adds a few seconds. |
| Subclustering | 2–30 minutes | Includes filtering + reclustering a subset of cells. (Whole analysis) |
| Correlation Network | 2–4 minutes | Larger clusters or using Kendall correlation may take longer. |
| GO Term Enrichment | 1–3 minutes | Depends on number of DE genes and ontology selected. |
| Pathway Analysis | 1–3 minutes | KEGG & Reactome databases processed similarly. |
| GSEA Analysis | 1–3 minutes | MSigDB categories vary in size; more permutations = longer time. |
| Cell-Cell Communication | 5–30 minutes | One of the longest steps. Time depends on the number of groups & PPI size. |
| Trajectory & Pseudotime | 3–30 minutes | UMAP-based; Monocle3 processing varies with complexity. |
| Co-expression Network (hdWGCNA) | 15 minutes to 1 hour | Metacell and soft-thresholding steps are the most time-consuming. |
| TF Regulatory Network | 30 minutes to 2 hours | Motif scanning + XGBoost modeling can be moderately slow. |

**Additional Notes:**

- Smaller datasets (<5k cells): Most steps complete in under 2–5 minutes.
- Larger datasets (>100k cells): Some modules may exceed 10 minutes to 2 hours.
- Most time-consuming modules:
  - JackStraw
  - Doublet Detection
  - SingleR
  - CellChat (Cell-Cell Communication)
  - Trajectory & Pseudotime
  - hdWGCNA
  - TF Regulatory Network
