# Supplementary figures and images for: ScRDAVis: An R shiny application for single-cell transcriptome data analysis and visualization
Source: PLoS Comput Biol. 2025 Nov 13;21(11):e1013721. doi: 10.1371/journal.pcbi.1013721 (PMC12626302; doi:10.1371/journal.pcbi.1013721)

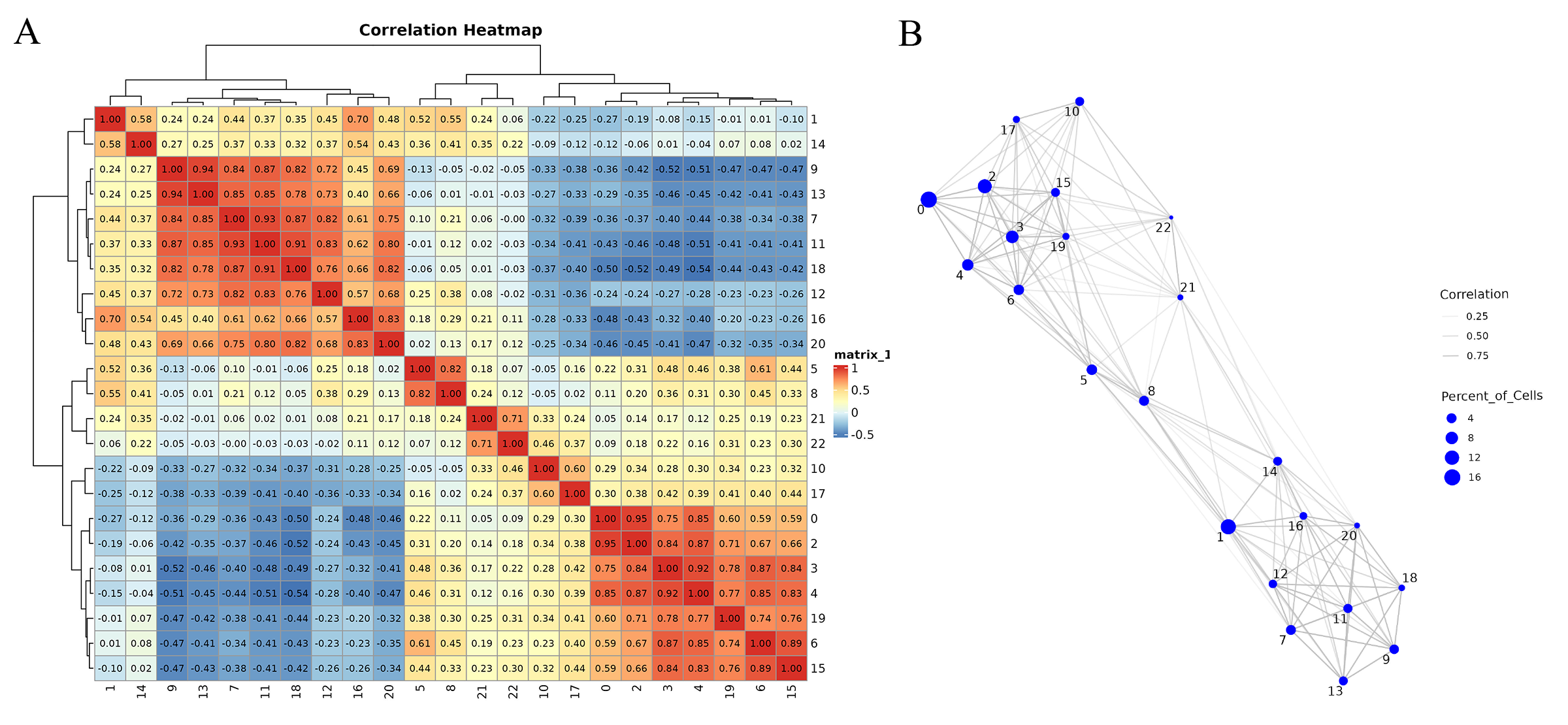

Supplement: S1 Fig — A) Heatmap depicting pairwise correlation values between the cell clusters. B) Correlation network plot between the cell clusters. (TIF) [file pcbi.1013721.s004.tif]

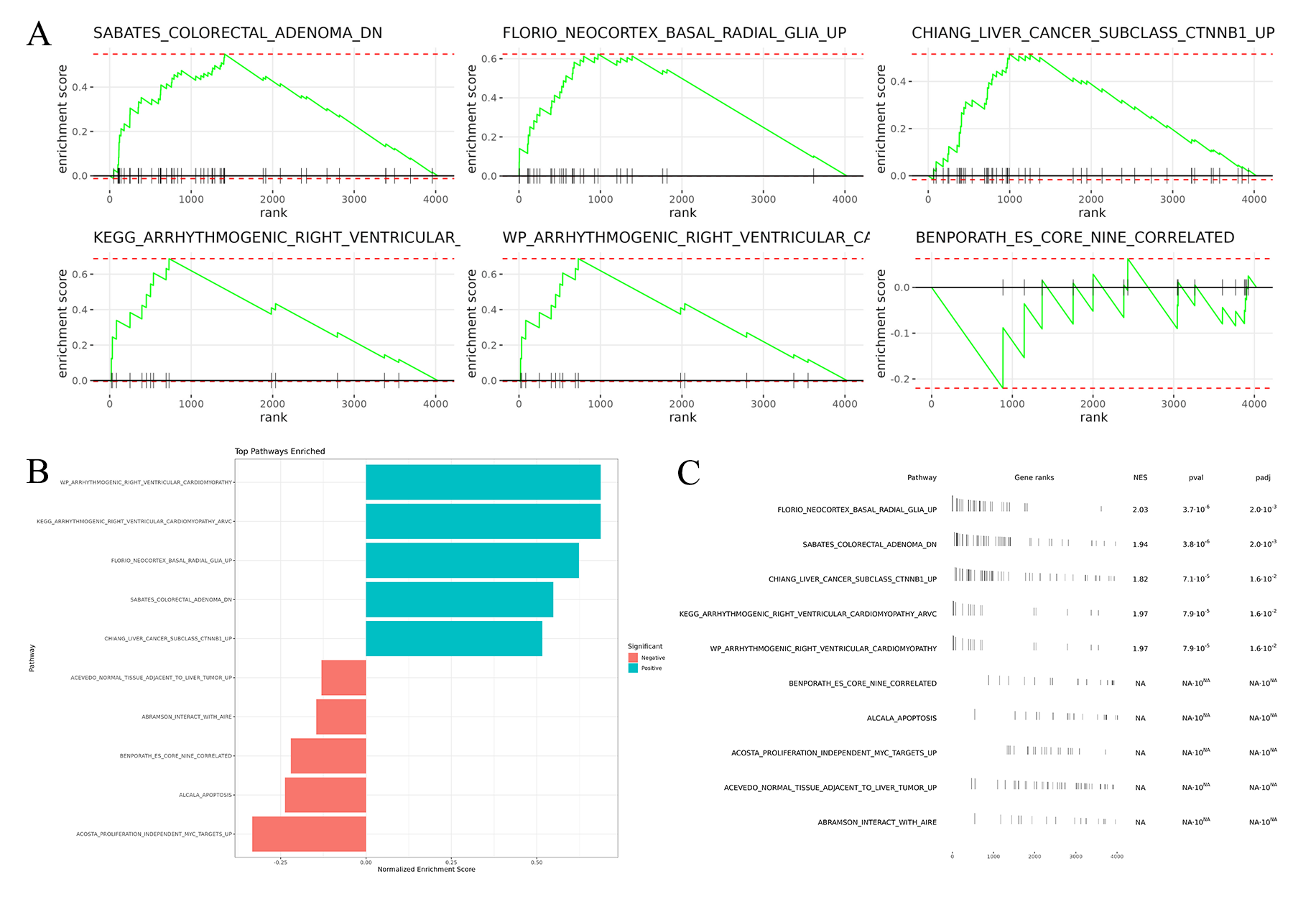

Supplement: S2 Fig — A) GSEA plot showing enrichment scores across ranked gene lists, highlighting leading-edge subsets. B) Bar plot summarizing the top enriched pathways ranked by significance. C) Table plot (PlotGseaTable) combining enrichment scores, p-values, and pathway annotations for detailed interpretation. (TIF) [file pcbi.1013721.s005.tif]
